# Mainlobe Interference Suppression Based on POL-SPICE and Covariance Matrix Reconstruction for Polarization-Sensitive Arrays

**DOI:** 10.3390/s26092604

**Published:** 2026-04-23

**Authors:** Buma Xiao, Huafeng He, Liyuan Wang, Tao Zhou

**Affiliations:** College of Missile Engineering, Rocket Force Engineering University, Xi’an 710025, China; bumaxiao@163.com (B.X.); hansw87@163.com (L.W.); ztxm99@163.com (T.Z.)

**Keywords:** mainlobe interference suppression, sparse iterative covariance-based estimation, covariance matrix reconstruction, joint spatial–polarization domain beamforming

## Abstract

Adaptive beamforming based on polarization-sensitive arrays enables joint spatial–polarization filtering for mainlobe interference suppression, but mainlobe distortion and performance degradation occur when the received data include the desired signal or multiple mainlobe interferences. Accordingly, this paper proposes a mainlobe interference suppression method based on Polarimetric Sparse Iterative Covariance-based Estimation (POL-SPICE) and covariance matrix reconstruction. This method utilizes the POL-SPICE algorithm to accurately estimate the direction of arrival (DOA), polarization, and power parameters. It reconstructs the covariance matrix by nulling the corresponding source power and constructs a feature projection matrix to preprocess the received signal. These eliminate the impact of the desired signal and mainlobe interference components on subsequent joint spatial–polarization domain beamforming, ultimately achieving interference suppression and mainlobe shape preservation. Simulation results illustrate that the proposed method is applicable to scenarios with the coexistence of the desired signal and multiple mainlobe interferences, and its superiority over existing methods is verified.

## 1. Introduction

With the rapid development of jamming technology, the electromagnetic environment faced by radar has become increasingly complex. Mainlobe jamming, characterized by flexible patterns, high similarity to target features, and difficulty in suppression, has become the preferred choice for jamming technology [1]. Adaptive beamforming technology forms nulls at the jamming location while retaining the desired signal by designing filter weight vectors. However, it will also form nulls within the mainlobe in the presence of mainlobe interference, causing distortion of the mainlobe and loss of target energy. Traditional mainlobe interference suppression techniques in the spatial domain mainly rely on two methods: Block Matrix Preprocessing (BMP) [2,3] and Eigen-Projection Matrix Preprocessing (EMP) [4,5]. These methods firstly block the mainlobe jamming and then combine beamforming to achieve jamming suppression. However, the BMP method requires accurate prior information about the mainlobe interference, and the EMP method needs to determine the eigenvector corresponding to the mainlobe jamming. Both methods rely on received data without the desired signal, which has certain limitations.

Adaptive beamforming based on polarization-sensitive arrays can utilize the differences in the spatial and polarization domains between interferences and the desired signal to form a joint spatial–polarization domain null in the direction of interference, offering more degrees of freedom and better interference suppression performance [6]. References [7,8] studied joint spatial–polarization domain beamforming algorithms and designed a step-size dynamic adjustment strategy to optimize algorithm performance. References [9,10] addressed the issues of mainlobe distortion and target energy loss caused by spatial–polarization domain null coupling when facing multiple mainlobe and sidelobe interferences in spatial–polarization domain adaptive beamforming. It proposed a multi-interference suppression method based on spatial–polarization domain null decoupling, which relies on received data without a desired signal and requires accurate estimation of interference parameters. Reference [11] proposed a two-step adaptive processing method and finally achieved interference suppression through Minimum Variance Distortion-less Response (MVDR) beamforming. References [12,13] proposed a phase-only adjustment method for polarization array transmit beamforming based on convex optimization, generating high-gain mainlobe and joint spatial–polarization domain nulls in specific polarization states. This method solves the problem of mainlobe distortion that traditional methods often cause when forming nulls in the interference region, but the phase relaxation iterative process requires multiple parameter adjustments, resulting in high computational complexity.

Most of the aforementioned methods rely on received data without desired signals. In practical scenarios, the severe degradation of beamforming performance caused by the presence of the desired signal component in the covariance matrix cannot be ignored. This issue is generally addressed through the reconstruction of the Interference-plus-Noise Covariance Matrix (INCM) to eliminate the influence of the desired signal component [14,15,16,17,18,19,20,21,22,23,24,25,26,27,28,29,30].

In terms of spatial domain signal processing, reference [14,15] pioneered a robust adaptive beamforming algorithm based on INCM reconstruction and desired signal steering vector estimation. This approach simultaneously addresses the issues of covariance matrix uncertainty and steering vector mismatch, providing a comprehensive theoretical framework for beamforming under mismatch and covariance contamination conditions. References [16,17] utilized the eigen-subspace theory as a foundation. Firstly, they reconstructed the INCM through power spectrum estimation and integration, then constructed an eigen-projection matrix and calculated the adaptive weight vector, achieving effective suppression of mainlobe interference. References [18,19] proposed a robust adaptive beamforming method based on steering vector (SV) estimation. It combined Minimum Variance Distortionless Response (Capon) power spectrum search results to calculate the interference SV, employed the least squares algorithm to solve for the noise covariance matrix, and then reconstructed the INCM and calculated the weight vector. To further enhance the reconstruction accuracy of the INCM, references [20,21] addressed the SV mismatch problem by formulating a convex optimization problem. They combined the spatial spectrum estimation and covariance matrix reconstruction to suppress mainlobe interference. Reference [22] introduced a Gaussian–Legendre Quadrature (GLQ)-based covariance matrix reconstruction method. It constructs a projection matrix to remove the desired signal from the received data, thereby improving the reconstruction accuracy of the INCM. References [23,24,25] respectively employed the Iterative Adaptive Algorithm (IAA), the Taylor Estimator, and the improved Sparse Iterative Covariance Estimation (SPICE) algorithm to estimate signal power for reconstructing the INCM. Subsequently, they filtered out the mainlobe interference component through the eigen-projection matrix and reconstructed the Sidelobe-Interference-plus-Noise Covariance Matrix (SINCM) to correct for noise perturbations, achieving simultaneous suppression of mainlobe and sidelobe interference. However, these methods determine the eigenvector corresponding to the mainlobe interference by assessing the correlation between the eigenvector of the mainlobe interference and the spatial steering vector of the desired signal. In the presence of multiple mainlobe interferences, the SV mismatch problem will arise, leading to a significant degradation in interference suppression performance.

In the field of joint spatial–polarization domain signal processing, reference [26] designed a robust MVDR beamforming weight vector by reconstructing INCM within the interference signal interval and estimating its SV within the desired signal interval. However, the performance of the algorithm relies on the reasonable division of the angular interval, and an overly narrow interval may not cover the true parameters. Reference [27] proposed a joint spatial–polarization adaptive beamforming algorithm based on a dual-polarization conformal array. Based on the Multiple Signal Classification (MUSIC) power spectrum estimation, combined with INCM reconstruction and subspace projection, it achieves suppression of interference with arbitrary polarization and spatial parameters. However, there is ambiguity in defining the uncertain region during the reconstruction of the interference covariance matrix. References [28,29] proposed robust mainlobe interference suppression methods in the joint spatial–polarization domain. They integrated and reconstructed INCM through MUSIC spectral estimation and utilized quadratic convex optimization constraints to design the methods, effectively suppressing interference with arbitrary spatial and polarization parameters. But these methods rely on convex optimization solutions, resulting in high computational complexity. Reference [30] proposed a redundant reduction-based grid-less sparse reconstruction beamforming method. It converts signal space sparsity into row sparsity of the power-scaled polarization matrix and then calculates the optimal beam weights through parameter estimation and INCM reconstruction.

Most of the aforementioned methods reconstruct INCM by estimating the power spectrum and integrating over interference angular sectors separated from the direction of the desired signal. This approach necessitates the division of angular sectors between the desired signal and interference, and the reconstruction accuracy is influenced by the power spectrum resolution and integration step size. The robustness of the final interference suppression performance needs to be further enhanced. In the research of robust interference suppression techniques, reference [31,32] investigated a robust interference suppression method based on array antenna pattern synthesis. By optimizing the array weight vector, it achieves mainlobe shape preservation and sidelobe interference suppression, providing an effective paradigm for solving robust interference suppression problems under array perturbation and mismatch conditions.

In summary, research on mainlobe interference suppression methods in the joint spatial–polarization domain is still insufficient; thus, further research is necessary. This paper addresses the issue that the desired signal component presents in received data by introducing the SPICE algorithm into the parameter estimation of polarization-sensitive arrays. Then, the INCM is reconstructed by zeroing the corresponding signal power, which improves the reconstruction accuracy. To address the difficulties in identifying the eigenvectors corresponding to interferences and severe beam mainlobe distortion under multiple mainlobe interferences, we select the eigenvectors associated with mainlobe interferences based on parameter estimation results and then construct an eigen-projection matrix to block the mainlobe interferences. Subsequently, we utilize the reconstructed SINCM to calculate the final spatial–polarization domain joint beamforming weight vector. By fully utilizing the polarization information differences between the target and interferences, effective suppression of mainlobe and sidelobe interferences is achieved while maintaining the shape of the mainlobe. The main contributions of this paper are as follows:

1. The DOA, polarization, and power parameters estimated by the POL-SPICE algorithm exhibit higher accuracy than those obtained by spectral estimation methods such as MUSIC and Capon;
2. To eliminate the influence of the desired signal component, we reconstruct the INCM by zeroing the desired signal power. To reduce the impact of eigen-projection preprocessing on subsequent beamforming, we reconstruct the SINCM by simultaneously zeroing the powers of both the desired signal and mainlobe interference;
3. By using the estimated mainlobe interference SV to select its corresponding eigenvectors rather than the desired signal SV adopted in conventional methods, more accurate eigenvectors associated with mainlobe interferences can be obtained for constructing the eigen-projection matrix. Additionally, the proposed method is robust to multiple mainlobe interferences.

## 2. Signal Model and Problem Formulation

**Figure 1:**
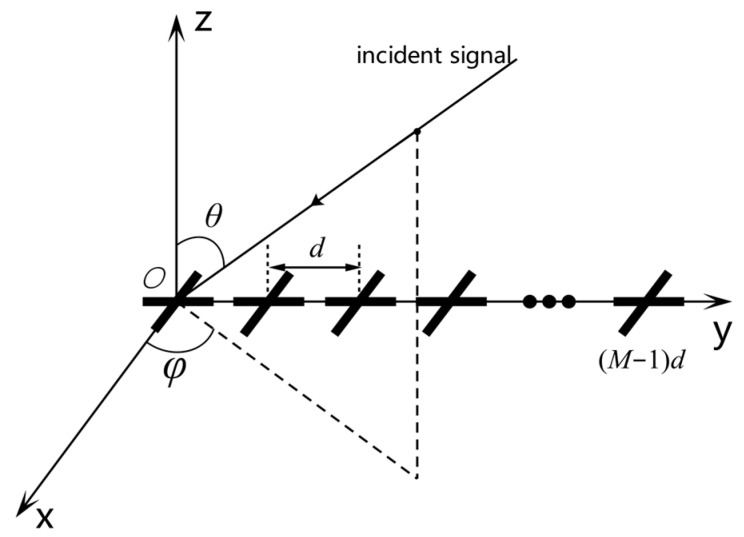
Uniform linear array composed of dual-orthogonal dipoles.

Consider a polarization-sensitive uniform linear array consisting of M orthogonal dipoles, where the orthogonal dipoles are placed along the x-axis and y-axis, with a spacing of d between the elements, as shown in Figure 1. Assuming that source i is incident on the array at elevation angle $\theta_{i}$ and azimuth angle $\varphi_{i}$, then its corresponding spatial steering vector is

(1) $$a_{s}(\theta_{i},\varphi_{i})={\left[e^{-j\phi_{1i}},e^{-j\phi_{2i}},\ldots ,e^{-j\phi_{Mi}}\right]}^{T},$$

where ${\left(\cdot \right)}^{T}$ is the transpose operation, $\phi_{mi}=\frac{2\pi (m-1)d\sin\theta_{i}\sin\varphi_{i}}{\lambda },\;m=1,2,\ldots ,M$ represents the phase delay of the ith element of the signal source m relative to the coordinate origin, and λ is the signal wavelength.

The polarization steering vector is related to the polarization state and spatial angle of the signal. For the dual-orthogonal dipoles, each element can receive electric field vectors in the x and y directions. Therefore, the polarization steering vector [33] corresponding to the signal source is

(2) $$a_{p}\left(\theta_{i},\varphi_{i},\gamma_{i},\eta_{i}\right)=\left[\begin{array}{cc} -\sin\varphi_{i} & \cos\theta_{i}\cos\varphi_{i}\\ \cos\varphi_{i} & \cos\theta_{i}\sin\varphi_{i} \end{array}\right]\left[\begin{array}{c} \cos\gamma_{i}\\ \sin\gamma_{i}e^{j\eta_{i}} \end{array}\right],$$

where $\gamma_{i}$ and $\eta_{i}$ are the polarization radiation angle and polarization phase angle of the signal, respectively. The joint spatial–polarization domain steering vector corresponding to this source is obtained as

(3) $$a(\theta_{i},\varphi_{i},\gamma_{i},\eta_{i})=a_{s}(\theta_{i},\varphi_{i})⊗a_{p}(\theta_{i},\varphi_{i},\gamma_{i},\eta_{i}),$$

where ⊗ is the Kronecker product. To simplify the writing, we let $a\left(\Theta_{i}\right)=a\left(\theta_{i},\varphi_{i},\gamma_{i},\eta_{i}\right)$. The received signal of the array is the superposition of the responses of each source. Assuming that there are q+r+1 far-field narrowband signals incident on the array, including 1 expected signal, q main lobe interferences, and r side lobe interferences, then the received signal model can be expressed as

(4) $$\begin{array}{ll} X\left(t\right) & =a\left(\Theta_{0}\right)s_{0}\left(t\right)+\sum_{i=1}^{q}a\left(\Theta_{i}\right)s_{i}\left(t\right)+\sum_{i=q+1}^{q+r}a\left(\Theta_{i}\right)s_{i}\left(t\right)+N\left(t\right)\\ & =AS\left(t\right)+N\left(t\right) \end{array}$$

where $A=\left[a\left(\Theta_{0}\right),a\left(\Theta_{1}\right),\ldots ,a\left(\Theta_{q}\right),\ldots ,a\left(\Theta_{q+r}\right)\right]$ denotes the steering vector matrix of the signal, $a(\Theta_{0})$ and $a(\Theta_{i})$ are the spatial–polarization domain joint steering vectors of the desired signal and each interference signal, respectively, $S\left(t\right)={\left[s_{0}\left(t\right),s_{1}\left(t\right),\ldots ,s_{q+r}\left(t\right)\right]}^{T}$ is the received signal vector, $s_{o}(t)$, $s_{i}(t)$ are the complex envelopes of the desired signal and each interference signal, respectively, and $N\left(t\right)={\left[n_{1}\left(t\right),n_{2}\left(t\right),\ldots ,n_{2M}\left(t\right)\right]}^{T}$ is a complex Gaussian white noise vector with zero mean and fixed variance.

Assuming that the desired signal, interferences, and noise are statistically independent of each other, the covariance matrix of the array received data can be theoretically expressed as

(5) $$\begin{array}{ll} R_{x} & =E\{X(t)X^{H}(t)\}\\ & =\sum_{i=0}^{q+r}p_{i}a(\Theta_{i})a^{H}(\Theta_{i})+\sigma I_{2M}\\ & =APA^{H}+\sigma I_{2M} \end{array}$$

where E{⋅} represents the expected value, ${\left(\cdot \right)}^{H}$ is the Hermitian transpose operation, and $p_{i}=E\left\{{\left|s_{i}\left(t\right)\right|}^{2}\right\}$ denotes the incident power of source i. This paper assumes that the noise variance received by each antenna element is identical and equal to σ. $I_{2M}$ represents the unit matrix of order 2M, $P=\mathrm{diag}\left(p_{0},p_{1},\ldots ,p_{q+r}\right)$ is the power matrix of the signal, and $\mathrm{diag}\left(\cdot \right)$ denotes the diagonal matrix.

Polarization-sensitive arrays can use adaptive beamforming technology to jointly utilize polarization domain and spatial domain information to suppress interference. The output of the beamformer can be obtained as

(6) $$y\left(t\right)=w^{H}X\left(t\right),$$

where $w={\left[w_{1},w_{2},\ldots ,w_{2M}\right]}^{T}$ is the joint filtering weight vector. According to the beamforming criterion, the traditional MVDR [18,19] criterion is extended to the joint spatial–polarization domain, which is named PMVDR in this paper. The beamforming weight vector is calculated as

(7) $$w=\frac{R_{in}^{-1}a\left(\Theta_{0}\right)}{a^{H}\left(\Theta_{0}\right)R_{in}^{-1}a\left(\Theta_{0}\right)},$$

where $R_{in}$ is the covariance matrix of interference and noise, which is difficult to obtain directly in the actual process. Usually, the sample covariance matrix ${\hat{R}}_{x}$ is used to replace $R_{in}$, that is

(8) $${\hat{R}}_{x}=\frac{1}{N}\sum_{n=1}^{N}X\left(n\right)X^{H}\left(n\right),$$

where N is the number of sampling points. It is worth noting that the data received by the array generally includes the desired signal, and the impact on beamforming performance can be ignored when the signal-to-noise ratio is low. But as the signal-to-noise ratio (SNR) increases, the deviation between ${\hat{R}}_{x}$ and $R_{in}$ increases, so directly using ${\hat{R}}_{x}$ to calculate the weight vector w will lead to a serious decline in the performance of beamforming. In references [22,25,26,27], the INCM is reconstructed to eliminate the influence of the desired signal component. The Capon power spectrum or MUSIC power spectrum estimators are usually used to integrate the INCM at the angle sector of the interference signal. The method of reconstructing the covariance matrix using Capon power spectrum integration can be expressed as

(9) $${\hat{R}}_{in}=\int_{\bar{\Omega }}P_{Capon}(\Theta )a(\Theta )a^{H}(\Theta )d\Theta ,$$

where $\Omega =[\Theta_{0}-\Delta \Theta_{0},\Theta_{0}+\Delta \Theta_{0}]$ represents the angular sector containing only the desired signal and $\bar{\Omega }$ represents the complementary sector of Ω. When mainlobe interference exists, it is also contained within the region $\bar{\Omega }$. The Capon spectrum is calculated as

(10) $$P_{Capon}\left(\Theta \right)=\frac{1}{a^{H}\left(\Theta \right){\hat{R}}_{x}^{-1}a\left(\Theta \right)},$$

Through analysis, we can obtain that the INCM reconstruction method relies on the estimation accuracy of the Capon power spectrum and the division of angle sectors. The resolution of this spectrum is limited, and its estimation accuracy decreases under low SNR conditions. In the presence of mainlobe interference, it is difficult to divide the angle sectors within the mainlobe, which limits its applicability. In addition, the MUSIC power spectrum has a high spectral resolution and is often used for INCM reconstruction, but it requires the number of sources to be known in advance and involves subspace decomposition operations.

After obtaining the covariance matrix that removes the desired signal component, the PMVDR method can generate joint null in the spatial–polarization domain in the interference direction. For mainlobe interference, interference suppression can be achieved by comprehensively utilizing the polarization information difference between the interference and the target. However, there is a coupling relationship between the interference null in this joint domain [10]. In the presence of multiple mainlobe interferences, it can also cause severe distortion of the mainlobe, resulting in the energy loss of the target.

## 3. Proposed Method

In this section, we proposed a mainlobe interference suppression method for polarization-sensitive arrays based on the POL-SPICE algorithm and covariance matrix reconstruction. Firstly, a polarization-sensitive array signal reception model is established. The POL-SPICE algorithm is utilized to estimate the direction of arrival, polarization, and power parameters. Subsequently, we calculate the steering vector of the signal based on the parameter estimation results. The INCM is reconstructed by setting the desired signal power to zero, and then its eigen-decomposition is performed to separate the interference subspace and noise subspace. The eigenvectors corresponding to the mainlobe interferences are filtered out based on the correlation between the steering vector of the mainlobe interference and its corresponding eigenvector. These eigenvectors are then used to construct an eigen-projection matrix, which will be utilized for preprocessing the received data. Next, the power of the mainlobe interference is set to zero to reconstruct the Sidelobe-Interference-plus-Noise Covariance matrix. Finally, we calculate the joint spatial–polarization beamforming weight and utilize it to weight the preprocessed data. Ultimately, effective suppression of both mainlobe and sidelobe interferences is achieved while preserving the shape of the mainlobe.

**Figure 2:**
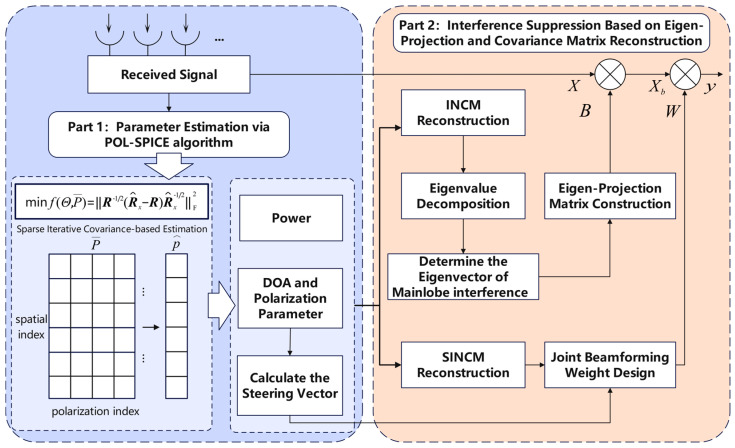
Flowchart of the proposed method.

The method flow of this paper is shown in Figure 2.

### 3.1. Parameter Estimation Based on POL-SPICE Algorithm

The SPICE algorithm is an adaptive sparse estimation algorithm without hyperparameters, which is based on covariance fitting criteria and can estimate the power of signals from useful data. It has superior performance in small snapshots and low signal-to-noise ratios and has global convergence [34,35,36]. This section applies the SPICE algorithm to the parameter estimation problem of polarization-sensitive arrays, known as the POL-SPICE method. The parameter estimation results provide a basis for subsequent INCM reconstruction.

Assuming that the signal of interest is distributed on a grid with discrete points L(L≫q+r+1), based on Equation (5), the covariance matrix of the received data can be expressed as

(11) $$R=U_{L}P_{L}U_{L}^{H}+\sigma I_{2M}≜U\bar{P}U^{H},$$

where

(12) $$\begin{array}{ll} U & =[U_{L},I_{2M}]\\ & =[a(\Theta_{1}),a(\Theta_{2}),\ldots ,a(\Theta_{L}),I_{2M}]\\ & ≜[u_{1},u_{2},\ldots ,u_{L},\ldots ,u_{L+2M}] \end{array}$$

and

(13) $$\begin{array}{ll} \bar{P} & =\left[\begin{array}{cc} P_{L} & 0\\ 0 & \sigma I_{2M} \end{array}\right]\\ & =\mathrm{diag}(p_{1},p_{2},\ldots ,p_{L},\sigma_{1},\ldots ,\sigma_{2M})\\ & ≜\mathrm{diag}(p_{1},p_{2},\ldots ,p_{L},p_{L+1},\ldots ,p_{L+2M}) \end{array}$$

where $U_{L}=\left[a\left(\Theta_{1}\right),a\left(\Theta_{2}\right),\ldots ,a\left(\Theta_{L}\right)\right]$ denotes the sparse array flow pattern dictionary matrix and $P_{L}=\mathrm{diag}\left(p_{1},p_{2},\ldots ,p_{L}\right)$ is the power diagonal matrix. It is worth noting that in this article, we only consider the case where the noise variance is the same, that is $\sigma_{1}=\cdots =\sigma_{2M}≜\sigma$. For the case where the noise variance is different, please refer to the reference [34].

Based on the SPICE algorithm, two-dimensional parameter and power estimation in the spatial–polarization domain can be achieved by minimizing the fitting performance indicator of the following covariance matrix, that is

(14) $$f\left(\Theta ,\bar{P}\right)={\left\|R^{-1/2}\left({\hat{R}}_{x}-R\right){\hat{R}}_{x}^{-1/2}\right\|}_{F}^{2},$$

where ${\left\|\cdot \right\|}_{F}$ denotes the Frobenius norm of the matrix, ${\hat{R}}_{x}$ is the sample covariance matrix, and $R^{-1/2}$ represents the positive definite square root of the Hermitian matrix $R^{-1}$. The SPICE algorithm approaches the optimal $\bar{P}$ infinitely by minimizing the covariance fitting criterion described in Equation (14). Through deduction, the minimization of function f is equivalent to the minimization of the following function:

(15) $$g\left(\Theta ,\bar{P}\right)=\mathrm{tr}\left({\hat{R}}_{x}^{1/2}R^{-1}{\hat{R}}_{x}^{1/2}\right)+\sum_{l=1}^{L+2M}\left(u_{l}^{H}{\hat{R}}_{x}^{-1}u_{l}\right)p_{l},$$

where $\mathrm{tr}\left(\cdot \right)$ represents the trace of the matrix. To solve this problem, we transform it into a constrained minimization problem, that is

(16) $$\underset{p_{l}⩾0}{\min}\mathrm{tr}\left({\hat{R}}_{x}^{1/2}R^{-1}{\hat{R}}_{x}^{1/2}\right)\;\;\;\;s.t.\sum_{l=1}^{L+2M}w_{l}p_{l}=1,$$

where $w_{l}=\frac{u_{l}^{H}{\hat{R}}_{x}^{-1}u_{l}}{2M}$. The optimization problem shown in Equation (16) is a semi-positive definite programming problem with high computational complexity. The SPICE algorithm seeks the optimal solution through iterative updates, and its iterative formulas are

(17) $$p_{l}^{i+1}=p_{l}^{i}\frac{{\left\|u_{l}^{H}R^{-1}\left(i\right){\hat{R}}_{x}^{1/2}\right\|}_{2}}{w_{l}^{1/2}\rho \left(i\right)},$$

(18) $$\sigma^{i+1}=\sigma^{i}\frac{{\left\|R^{-1}\left(i\right){\hat{R}}_{x}^{1/2}\right\|}_{2}}{\mu^{1/2}\rho \left(i\right)},$$

where, ${\left\|\cdot \right\|}_{2}$ represents the 2-norm of the matrix, index i denotes the number of iterations, $R\left(i\right)$ can be iteratively updated through Equation (11), and

(19) $$\mu =\sum_{l=L+1}^{L+2M}w_{l},$$

(20) $$\rho \left(i\right)=\sum_{l=1}^{L}w_{l}^{1/2}p_{l}^{i}{\left\|u_{l}^{H}R^{-1}\left(i\right){\hat{R}}_{x}^{1/2}\right\|}_{2}+\mu^{1/2}\sigma^{i}{\left\|R^{-1}\left(i\right){\hat{R}}_{x}^{1/2}\right\|}_{2}$$

The algorithm can be initialized with the power estimates obtained by means of the periodogram method:

(21) $$p_{l}^{0}=\frac{u_{l}^{H}{\hat{R}}_{x}u_{l}}{{\left\|u_{l}\right\|}_{F}^{4}},\;\;\sigma^{0}=\min p_{l}^{0}$$

In practical applications, the algorithm requires presetting a maximum number of iterations and a convergence threshold to avoid endless iterations. We define a convergence threshold $r_{p}$ as the relative rate of change in signal power between adjacent iterations, that is

(22) $$r_{p}=\frac{{\left\|{\hat{p}}_{L}^{i+1}-{\hat{p}}_{L}^{i}\right\|}_{F}}{{\left\|{\hat{p}}_{L}^{i}\right\|}_{F}},$$

where ${\hat{p}}_{L}^{i}=\left[p_{1}^{i},p_{2}^{i},\ldots ,p_{L}^{i}\right]$ is the estimated signal power vector for each iteration. When the relative rate of change in power is less than or equal to the convergence threshold, the algorithm stops iterating.

Finally, the power spectrum of the signal can be estimated from Equation (17), and the DOA and polarization parameters of each source are determined by the peak positions and values of the power spectrum, which are denoted as ${\hat{\Theta }}_{0},{\hat{\Theta }}_{1},\ldots ,{\hat{\Theta }}_{q+r}$, along with the signal power ${\hat{p}}_{0},{\hat{p}}_{1},\ldots ,{\hat{p}}_{q+r}$ corresponding to each source.

Unlike the MUSIC algorithm, the SPICE algorithm does not require estimation of the number of sources, and the obtained power spectrum has the characteristics of sparse smoothness. In the absence of prior information, the accuracy of parameter estimation is less affected, making it suitable for resolving small angle intervals within the mainlobe range to cope with the presence of multiple mainlobe interferences.

### 3.2. INCM Reconstruction and Eigen-Projection Matrix Preprocessing

After estimating the DOA, polarization, and power parameters of the signal through the POL-SPICE algorithm, in order to suppress the mainlobe interference in a targeted manner, it is necessary to distinguish between main and side lobe interference and calculate their corresponding steering vectors based on the parameter estimation results. The calculation formula for the mainlobe width of a uniform linear array is

(23) $$BW_{0}=2\mathrm{arc}\sin\left(\frac{\lambda }{Md}+\sin\delta_{s}\right),$$

where $\delta_{s}$ is the DOA of the desired signal, and then the angle sector of the mainlobe can be divided into $\Phi =\left[\delta_{s}-\frac{BW_{0}}{2},\delta_{s}+\frac{BW_{0}}{2}\right]$, so combined with the estimation results of the POL-SPICE algorithm, it is easy to determine the main and side lobe interference and their corresponding steering vectors and power.

When the training data contains the desired signal, INCM reconstruction is usually achieved using methods based on power spectrum integration reconstruction, such as the MUSIC power spectrum [37], Capon power spectrum [38], etc., to eliminate the influence of the desired signal component. However, under the conditions of low SNR or the presence of multiple mainlobe interferences, the estimation accuracy of the power spectrum decreases and the reconstruction accuracy of the covariance matrix decreases; then, the interference suppression performance is affected. Unlike integral reconstruction methods, this paper uses the results of POL-SPICE parameter estimation to directly reconstruct the covariance matrix by replacing the corresponding values of the power diagonal matrix. There is no need to integrate in a specific angle region, reducing the signal processing flow. The reconstructed covariance matrix has higher accuracy and stronger robustness.

To remove the desired signal component, we reset the desired signal power to zero to reconstruct INCM, that is

(24) $${\hat{R}}_{in}=\hat{A}P_{1}{\hat{A}}^{H}+\hat{\sigma }I_{2M},$$

where $\hat{A}=\left[a\left({\hat{\Theta }}_{0}\right),a\left({\hat{\Theta }}_{1}\right),\ldots ,a\left({\hat{\Theta }}_{q+r}\right)\right]$ represents the calculated signal steering vector matrix, $P_{1}=\mathrm{diag}\left(0,{\hat{p}}_{1},{\hat{p}}_{2}\ldots ,{\hat{p}}_{q+r}\right)$ is the reconstructed power diagonal matrix, and $\hat{\sigma }$ denotes the estimation of noise power, which is obtained by taking the average of the non-target and interference eigenvalues after eigen-decomposing the original received data sample covariance matrix ${\hat{R}}_{x}$. This approach can reduce the impact of noise disturbances, that is

(25) $$\hat{\sigma }=\frac{\lambda_{q+r+1}+\lambda_{q+r+2}+\ldots +\lambda_{2M}}{2M-q-r}$$

After obtaining the reconstructed INCM, it can be directly substituted into Equation (7) for joint spatial–polarization beamforming to achieve interference suppression. However, in the presence of multiple mainlobe interferences, multiple notches will be formed in the mainlobe during beamforming, which can also cause mainlobe distortion and reduce the target received power. To this end, this paper combines the spatial anti-mainlobe interference algorithm: Eigen-Projection Matrix Preprocessing and covariance matrix reconstruction (EMP-CMR) [4,5]. It firstly constructs an eigen-projection matrix to block the mainlobe interference, then sets the power of the mainlobe interference to zero to reconstruct SINCM, and finally performs joint adaptive beamforming to suppress main and side lobe interference while reducing mainlobe distortion. By performing an eigen-decomposition on the covariance matrix reconstructed from Equation (24), we can obtain

(26) $${\hat{R}}_{in}=\sum_{i=1}^{2M}\lambda_{i}e_{i}e_{i}^{H}=E_{s}\Lambda_{s}E_{s}^{H}+E_{n}\Lambda_{n}E_{n}^{H},$$

where $\lambda_{i}$ is the ith eigenvalue of ${\hat{R}}_{in}$, $e_{i}$ denotes its corresponding eigenvector, and the eigenvalues are sorted in descending order: $\lambda_{1}⩾\lambda_{2}⩾\cdots ⩾\lambda_{q+r}⩾\cdots ⩾\lambda_{2M}$. $E_{s}=\left[e_{1},e_{2},\ldots ,e_{q+r}\right]$ is the interference subspace, $\Lambda_{s}=\mathrm{diag}\left[\lambda_{1},\lambda_{2},\ldots ,\lambda_{q+r}\right]$ is its corresponding power matrix, $E_{n}=\left[e_{q+r+1},e_{q+r+2},\ldots ,e_{2M}\right]$ represents the noise subspace, and $\Lambda_{n}=\mathrm{diag}\left[\lambda_{q+r+1},\lambda_{q+r+2},\ldots ,\lambda_{2M}\right]$.

Assuming that $E_{m}=\left[e_{m1},e_{m2},\ldots ,e_{mq}\right]$ is a matrix composed of the eigenvectors corresponding to the multiple mainlobe interferences, the eigen-projection matrix can be constructed as

(27) $$B=I_{2M}-E_{m}{\left(E_{m}^{H}E_{m}\right)}^{-1}E_{m}^{H}$$

The above process steps also require determining the eigenvector corresponding to each mainlobe interference. In practical applications, the power of main and side lobe interference often exhibits non-uniform distribution characteristics and weak spatial correlation. Therefore, the subspace formed by the mainlobe interference steering vector and its corresponding eigenvector can be approximately equivalent [24]. Within the mainlobe, the spatial correlation between multiple sources is relatively high. Traditional methods select the eigenvectors corresponding to mainlobe interferences based on the correlation between the steering vector of the desired signal and the interference eigenvectors. In the presence of multiple mainlobe interferences, this approach easily leads to mismatch problems.

**Figure 3:**
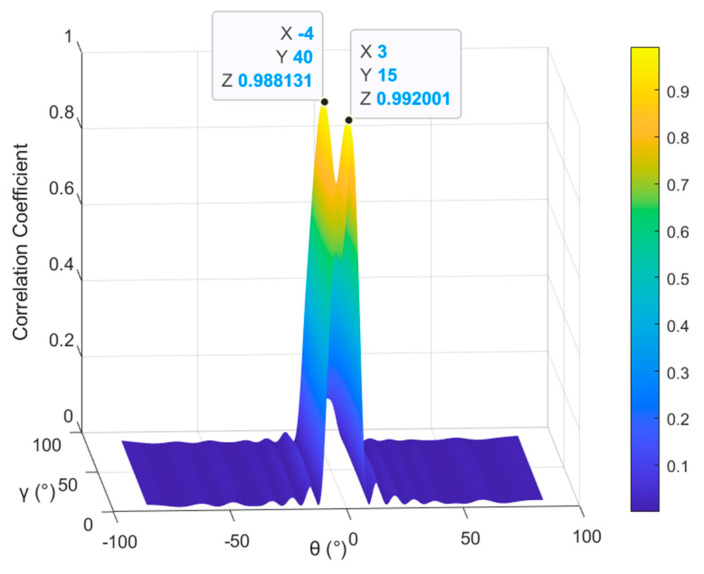
Subspace response of the eigenvector corresponding to mainlobe interferences.

Thanks to the parameter estimation results of the POL-SPICE algorithm, the steering vector of the mainlobe interference can be calculated. Due to the correlation between the steering vector of the mainlobe interference and its corresponding eigenvector $e_{m}$ being the strongest, the correlation coefficient method is used to determine the eigenvectors corresponding to multiple mainlobe interferences, and the judgment criterion is

(28) $$\left|\rho \left(e_{mk},a\left({\hat{\Theta }}_{k}\right)\right)\right|=\underset{e_{i}}{\max}\left|\rho \left(e_{i},a\left({\hat{\Theta }}_{k}\right)\right)\right|,$$

where 1⩽i⩽q+r, 1⩽k⩽q, and $\rho \left(v_{1},v_{2}\right)=v_{1}^{H}v_{2}/\left(\left\|v_{1}\right\|\left\|v_{2}\right\|\right)$ represents the correlation coefficient between any two vectors $v_{1}$ and $v_{2}$ and $a({\hat{\Theta }}_{k})$ is the estimated mainlobe interference steering vector. To intuitively characterize the selection mechanism of eigenvectors corresponding to multiple mainlobe interferences, we configure a scenario with two mainlobe interferences (parameter settings are given in Table 1). We calculate the correlation coefficients between the mainlobe interference eigenvectors and the estimated steering vectors of each interference, namely the subspace responses of the mainlobe interference eigenvectors. The simulation results are shown in Figure 3.

The two peak values in the figure correspond to the correlation coefficients between the two mainlobe interferences within the mainlobe and their corresponding eigenvectors, respectively. The peak values are close to 1, while the values in other directions approach 0, indicating that the proposed selection method can effectively identify the eigenvectors corresponding to each mainlobe interference, thus ensuring the accuracy of the constructed eigen-projection matrix.

Then, the eigen-projection matrix B is used to preprocess the echo data, where the mainlobe interference component is effectively suppressed and the desired signal and other side lobe interference are retained. Finally, the preprocessed data is

(29) $$X_{b}\left(t\right)=BX\left(t\right)$$

### 3.3. SINCM Reconstruction and Adaptive Beamforming

After the eigen-projection preprocessing, the suppression of mainlobe interference may not be complete. Traditional minimum variance undistorted response beamforming still exhibits mainlobe distortion in residual mainlobe interference scenarios, and in multiple mainlobe interference situations, the array response in the direction of sidelobe interference increases, and the interference null becomes shallower. Based on this, we reset the mainlobe interference power to zero to reconstruct SINCM, eliminating the influence of the mainlobe interference component completely, that is

(30) $$R_{rec}=\hat{A}P_{2}\hat{A}+\hat{\sigma }I_{2M},$$

where $P_{2}=\mathrm{diag}\left(0,\ldots ,0,{\hat{p}}_{q+1},\ldots ,{\hat{p}}_{q+r}\right)$ is the reconstructed power diagonal matrix.

Then the final adaptive weight vector can be obtained as

(31) $$w=\frac{R_{rec}^{-1}a\left(\Theta_{0}\right)}{a^{H}\left(\Theta_{0}\right)R_{rec}^{-1}a\left(\Theta_{0}\right)}$$

Through the above eigen-projection preprocessing and SINCM reconstruction process, the mainlobe interference has been suppressed, and no null will be formed in the mainlobe during beamforming, achieving mainlobe shape preservation and sidelobe interference suppression. The final output of beamforming is

(32) $$y\left(t\right)=w^{H}X_{b}\left(t\right)=w^{H}BX\left(t\right)$$

### 3.4. Performance Analysis

(A) Output SINR Analysis

The performance of traditional spatial interference suppression methods significantly decreases in the presence of mainlobe interference. For polarization-sensitive arrays, as long as there is a certain difference in their polarization states, beamforming can still effectively suppress interference even if the interference is in the same direction as the target. The method proposed in this paper not only achieves the shape preservation effect of the mainlobe but also has deeper sidelobe interference suppression notches. The signal-to-noise ratio (SINR) of the array output is calculated as

(33) $$\mathrm{SINR}=\frac{w^{H}R_{s}w}{w^{H}R_{in}w},$$

where $R_{s}=p_{0}a\left(\Theta_{0}\right)a^{H}\left(\Theta_{0}\right)$ represents the covariance matrix of the desired signal and the optimal output SINR is ${\mathrm{SINR}}_{opt}=p_{0}a^{H}\left(\Theta_{0}\right)R_{in}^{-1}a\left(\Theta_{0}\right)$. For the eigen-projection preprocessing methods, the covariance matrix after projection processing is $R_{b}=BR_{in}B^{H}$, and then the output SINR calculation formula becomes

(34) $$\mathrm{SINR}=\frac{w^{H}BR_{s}B^{H}w}{w^{H}BR_{in}B^{H}w}$$

(B) Computational Complexity Analysis

By summarizing the algorithmic procedure of the proposed method, its computational complexity mainly consists of three parts: the parameter estimation process based on the POL-SPICE algorithm, with a complexity of $O(NM^{2}+M^{3}CL+MCL^{2})$; the reconstruction and decomposition of the covariance matrix, with a computational complexity of $O(M^{3})$; and the derivation of the eigen-projection operator, with a computational complexity of $O(M^{2}q)$. Accordingly, the overall computational complexity of the proposed method is $O(NM^{2}+M^{3}CL+MCL^{2}+M^{3}+M^{2}q)$, where N,M,L denote the number of snapshots, the number of array elements, and the number of grids for spectral peak search, respectively; C is the preset number of iterations of the POL-SPICE algorithm; and q is the number of mainlobe interferences.

## 4. Simulation Results

In this section, the effectiveness of the method proposed in the article is verified through numerical simulation. We consider a polarization-sensitive uniform linear array consisting of 16 orthogonal dipoles, with element spacing of half a wavelength. Without loss of generality, this paper assumes that the interference signal and the desired signal are independent of each other, the noise is additive Gaussian white noise, and the received signals are all far narrowband field signals. For the convenience of displaying simulation results, the azimuth angle and polarization phase angle of all signal parameters are fixed at 90°, that is $\left(\varphi ,\eta \right)=\left(90{}^{\circ},90{}^{\circ}\right)$. Assuming that there is one desired signal and four interference signals incident on the array, including two mainlobe interferences and two sidelobe interferences, the angle parameters, SNR, and interference-to-noise ratio (INR) settings are shown in Table 1.

In order to analyze the simulation results, the proposed method was compared with PMVDR [8], INCM-PMVDR [26], EMP and EMP-CMR [4,5] algorithms. It is worth noting that EMP and EMP-CMR algorithms belong to conventional spatial mainlobe interference suppression methods, and their received data does not contain the desired signal. This paper extends them to the spatial–polarization joint domain for method comparison.

### 4.1. Example 1: Convergence of POL-SPICE Algorithm

In the POL-SPICE algorithm, we assumed that the spatial–polarization parameter $\left(\theta ,\gamma \right)$ is uniformly discretized into a set of grids, that is $\Theta =[\Theta_{1},\Theta_{2},\ldots ,\Theta_{L}]$, where the scanning grids are set to $\theta =\left[-90,90\right]$ and $\gamma =\left[0,90\right]$, the number of snapshots is set to N=256, the increment between adjacent grid points is 0.5°, the maximum iteration number is set to 100, and the convergence threshold is set to $r_{p}=10^{-4}$.

Firstly, we verified the convergence performance of the POL-SPICE algorithm by setting SNR to 10 dB and consider the simultaneous presence of two mainlobe interferences and two sidelobe interferences.

**Figure 4:**
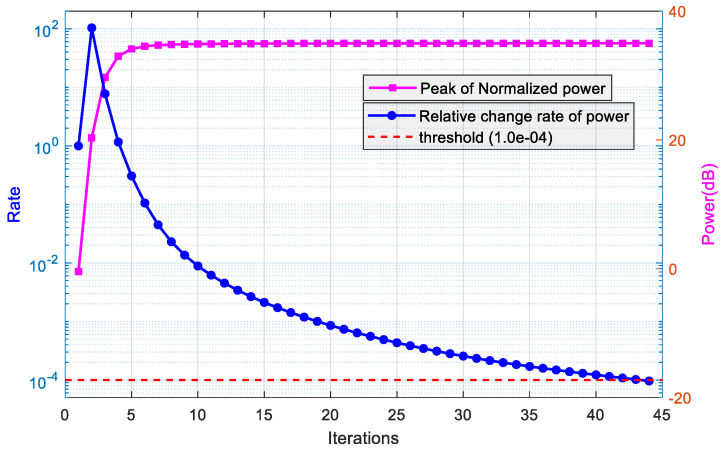
Convergence performance curve of the POL-SPICE algorithm.

Figure 4 shows the iterative performance of the POL-SPICE algorithm. Among them, the blue curve (left axis of coordinates) represents the convergence of the POL-SPICE algorithm, and the relative rate of power change gradually decreases with the number of iterations, ultimately reaching the convergence threshold level (red dashed line). The pink curve (right axis of the coordinate) shows the convergence speed of the algorithm. The estimated peak signal power increases with the number of iterations and tends to stabilize, indicating that the power gradually focuses on the true $\left(\theta ,\gamma \right)$. From the graph, it can be seen that the peak power starts to stabilize after about 15 iterations, indicating that the convergence speed of the algorithm is fast in the early stage.

### 4.2. Example 2: Estimation Performance of Joint Spectrum

**Figure 5:**
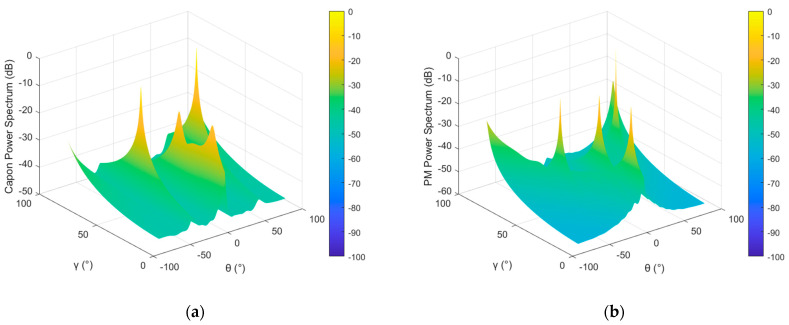

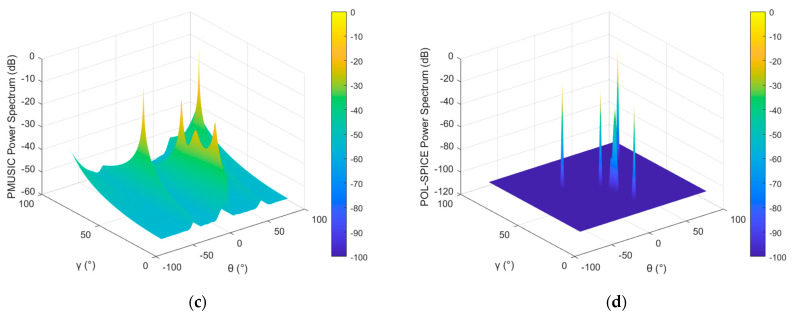
Joint spectrum in the case of two mainlobe interferences: (**a**) Capon algorithm; (**b**) PM algorithm; (**c**) MUSIC algorithm; (**d**) the proposed method.

In order to compare and analyze the parameter estimation performance of the POL-SPICE algorithm under multiple mainlobe interference conditions, the conventional power spectrum estimation algorithms MUSIC [37], Capon [38], Propagator Method [39] (PM) were extended to the parameter estimation problem of polarization-sensitive arrays, and their search grid accuracy was set to 0.5°, while the grid accuracy of the POL-SPICE algorithm remained unchanged. Figure 5 shows the joint spatial–polarization spectral estimation results of these four spectral estimation algorithms.

By analyzing the simulation results, it can be concluded that both the Capon algorithm and PM algorithm can only estimate four sources and cannot distinguish the desired signal with the lowest power. This is because there are many sources within the mainlobe, and the correlation between the sources increases, masking the desired signal with the lowest power. At the same time, there is also a certain error in power estimation. The MUSIC algorithm can distinguish all sources, but the power estimation error is large, which will affect the reconstruction accuracy of the covariance matrix. The estimation performance of the algorithm in this article is the best. The algorithm essentially belongs to the grid search method, with higher accuracy and obvious peak values in the power spectrum, without the need to estimate the number of sources.

We next calculate the Root-Mean-Square Error (RMSE) of the parameter estimation results to further verify the performance of the POL-SPICE algorithm. Since the Capon algorithm and PM algorithm can no longer effectively distinguish the sources, we select the Reduced-Dimensional MUSIC algorithm [40] (RD-MUSIC) as a supplementary comparative method. The RD-MUSIC algorithm can reduce the dimension of spectral peak search while guaranteeing the array resolution performance and estimation accuracy and does not require extra parameter pairing operations. The RMSE in this paper is defined as

(35) $$\mathrm{RMSE}=\sqrt{\frac{1}{M_{c}K}\sum_{m=1}^{M_{c}}\sum_{k=1}^{K}{\left({\hat{\alpha }}_{k,m}-\alpha_{k}\right)}^{2}},$$

where $M_{c}$ denotes the number of Monte-Carlo trials, K represents the total number of sources, and ${\hat{\alpha }}_{k,m}$ denotes an arbitrary element in the kth source parameter $\left({\hat{\theta }}_{k},{\hat{\varphi }}_{k},{\hat{\gamma }}_{k},{\hat{\eta }}_{k}\right)$ estimated from the mth Monte Carlo simulation.

**Figure 6:**
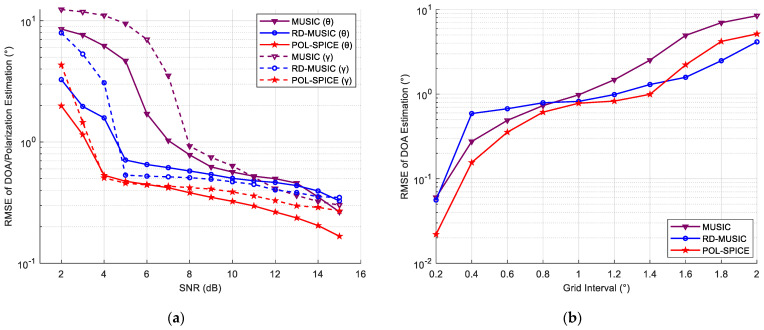
RMSE curve versus: (**a**) SNR; (**b**) grid interval.

Figure 6a shows the RMSE of the parameter estimation results obtained by the proposed algorithm, MUSIC, and RD-MUSIC over 50 Monte Carlo trials under different SNRs. As the SNR increases, the RMSE values of all these methods decrease gradually. The proposed method achieves the smallest RMSE under low SNR conditions and exhibits the best overall estimation performance. Tests indicate that the algorithm requires more iterations to stabilize its estimation performance at an even lower SNR.

Similar to the MUSIC and RD-MUSIC algorithms, the POL-SPICE algorithm is a grid-search-based method, so it is necessary to analyze its sensitivity to grid resolution and discretization errors. To this end, we set the grid interval from 0.2° to 2° and calculated the RMSE of the proposed method, with the results shown in Figure 6b. As the grid interval increases, the RMSE of all methods rises. Under the same grid conditions, the RMSE curves of different methods are generally close to each other, and the proposed method relies on the accuracy of grid division. Table 2 presents the comparison results of computational complexity among MUSIC, Capon, PM, RD-MUSIC and the proposed algorithm:

where N,M,L denote the number of snapshots, the number of array elements, and the number of grids for spectral peak search, respectively; C is the preset number of iterations of the POL-SPICE algorithm; $L^{\prime }$ denotes the number of grid search points after dimension reduction in the RD-MUSIC algorithm; and K represents the total number of sources.

Since the proposed method requires iterative searching, its computational complexity is higher than that of other methods. However, the core of the proposed method lies in obtaining accurate DOA, polarization, and power estimates via the POL-SPICE algorithm to achieve precise INCM reconstruction, which cannot be fulfilled by other methods. Therefore, at the cost of partial computation time, the proposed algorithm ensures high parameter estimation accuracy and is more suitable for scenarios with multiple mainlobe interferences.

### 4.3. Example 3: Effectiveness of Interference Suppression and Mainlobe Shape Preservation

After demonstrating the effectiveness of the parameter estimation algorithm proposed in this article, we tested the interference suppression capability of the proposed method. The joint spatial–polarization domain beam pattern of the array reflects the array response of beamforming weight vectors to signals with different incoming and polarization parameters. We defined it as

(36) $$F\left(\theta ,\varphi ,\gamma ,\eta \right)=\frac{{\left|w^{H}a\left(\theta ,\varphi ,\gamma ,\eta \right)\right|}^{2}}{\max{\left|w^{H}a\left(\theta ,\varphi ,\gamma ,\eta \right)\right|}^{2}}$$

**Figure 7:**
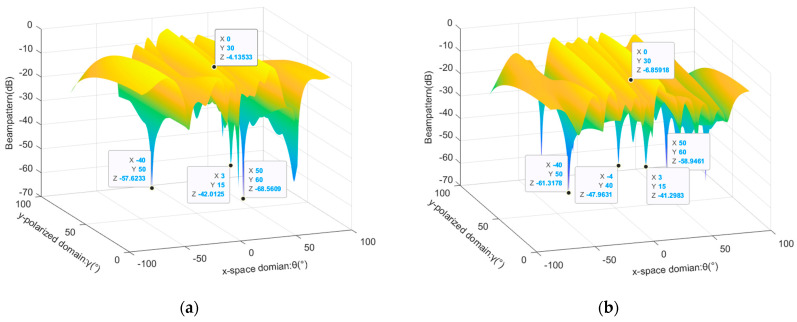

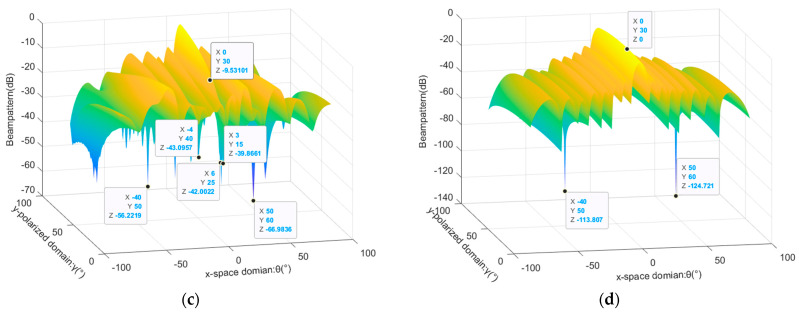
Comparison of joint beam pattern in the case of one or multiple mainlobe interferences: (**a**) PMVDR method in the case of one mainlobe interference; (**b**) PMVDR method in the case of two mainlobe interferences; (**c**) PMVDR method in the case of three mainlobe interferences; (**d**) the proposed method in the case of three mainlobe interferences.

Firstly, the joint beam patterns of PMVDR and the method proposed in this paper are compared, considering the presence of single mainlobe interference, two mainlobe interferences, and three mainlobe interferences. The added mainlobe interference parameter is set to $\left(\theta ,\gamma \right)=(6{}^{\circ},25{}^{\circ})$, INR=20 dB, and the other experimental conditions remain unchanged. The results are shown in Figure 7.

According to the graph analysis, the PMVDR method can utilize the polarization information difference between the interference and target to generate null in the polarization domain. However, there are also problems with mainlobe distortion and offset. Under the condition of three mainlobe interferences, the beam pattern gain at the target is reduced to −9.53 dB. This is because the PMVDR method directly performs adaptive beamforming based on the covariance matrix containing the desired signal. Because of the influence of the desired signal component and multiple mainlobe interferences, the performance of beamforming is reduced, resulting in energy loss of the target. The method proposed in this article greatly reduces the distortion of the mainlobe and improves the output SINR through eigen-projection preprocessing and the SINCM reconstruction process. Meanwhile, observing the suppression of sidelobe interference in the figure, the sidelobe level of the proposed method is lower, with deeper interference notches and better interference suppression effects.

**Figure 8:**
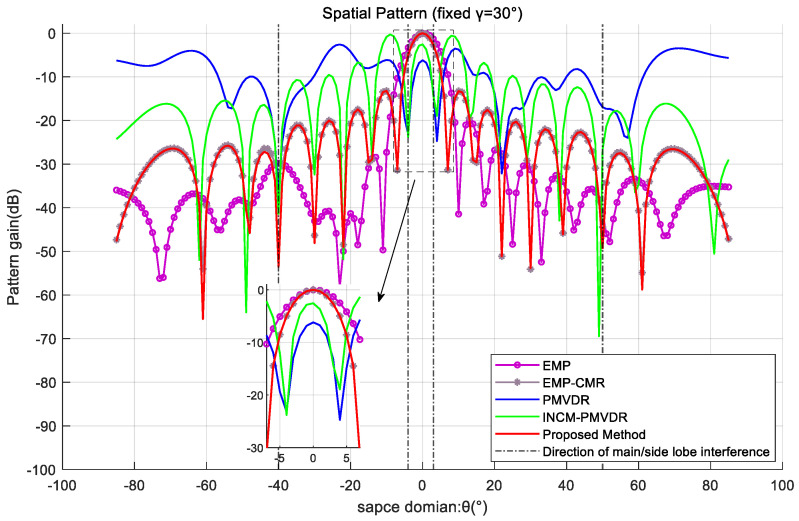
Spatial pattern at a fixed polarization angle profile in the case of two mainlobe interferences.

**Figure 9:**
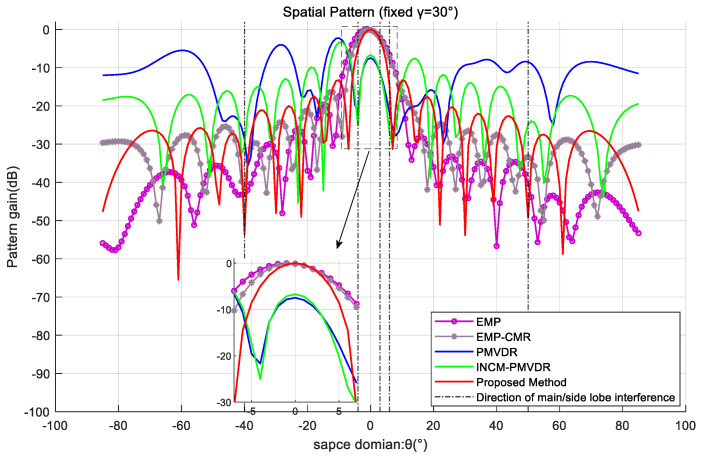
Spatial pattern at a fixed polarization angle profile in the case of three mainlobe interferences.

In order to further demonstrate the mainlobe shape preservation effect of the proposed method under multiple mainlobe interferences, the spatial beam pattern under a fixed polarization parameter profile was used for comparative analysis. By analyzing the profile with a fixed target polarization co-angle γ=30°, the beamforming effects of various methods are exhibited. The results are shown in Figure 8 and Figure 9.

The simulation results demonstrate that the method proposed in this paper exhibits optimal mainlobe shape preservation and low sidelobe level performance. In the presence of the desired signal and multiple mainlobe interferences, the PMVDR and INCM-PMVDR methods suffer from severe mainlobe deformation, deteriorating performance, and an overall increase in sidelobe levels, resulting in energy loss. The EMP method exhibits mainlobe offset and unsatisfactory sidelobe nulling performance, which results from performing beamforming directly on the eigen-projection preprocessed data. The EMP-CMR method also achieves favorable mainlobe shape preservation. But the two methods are implemented using received data that does not contain the desired signal. The proposed method achieves mainlobe shape preservation comparable to that of the EMP-CMR method while delivering superior sidelobe interference suppression performance, which verifies the effectiveness of the proposed method.

### 4.4. Example 4: Performance of Output SINR

**Figure 10:**
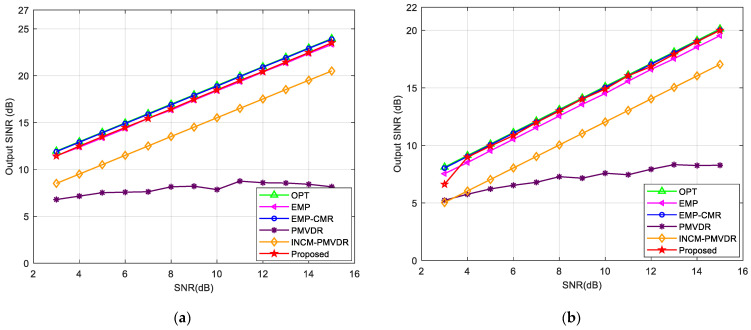
Output SINR versus input SNR in the case of: (**a**) one mainlobe interference; (**b**) multiple mainlobe interferences.

Next, we tested the output SINR performance of proposed method in the presence of the desired signal and multiple mainlobe interferences. We conducted 100 Monte Carlo simulations to analyze the relationship between output SINR, input SNR, and number of snapshots. We compared it with the optimal SINR output (OPT), EMP, EMP-CMR, PMVDR, and INCM-PMVDR methods and set single mainlobe and double mainlobe interference scenarios in simulation experiments. Firstly, we analyzed the variation in the output SINR of the proposed method with the input SNR. The number of snapshots was fixed at 256. Figure 10 shows the variation curves of the output SINR with the input SNR under single or multiple mainlobe interferences.

The results show that the output performance of the proposed method is superior to that of the EMP, PMVDR, and INCM-PMVDR methods. This is attributed to more accurate steering vector estimation and covariance matrix reconstruction accuracy. The output SINR increases linearly as the SNR increases and is closer to the theoretical optimal value. Only when the SNR is extremely low, the algorithm output is unstable. However, in the presence of oppressive interference, the impact of the desired signal component on beamforming performance can be ignored when the input SNR is extremely low, and there is no need to eliminate the influence of the desired signal component through INCM reconstruction. Due to the influence of the desired signal component that persists during beamforming, the output SINR of the PMVDR method increases slowly even as the SNR continues to increase. The INCM-PMVDR method is limited by the impact of angle sector partitioning and power estimation accuracy on reconstruction accuracy, resulting in a lower output SINR compared to the proposed method. Meanwhile, under the scenarios of multiple mainlobe interferences, the proposed method can maintain performance close to the optimal output.

**Figure 11:**
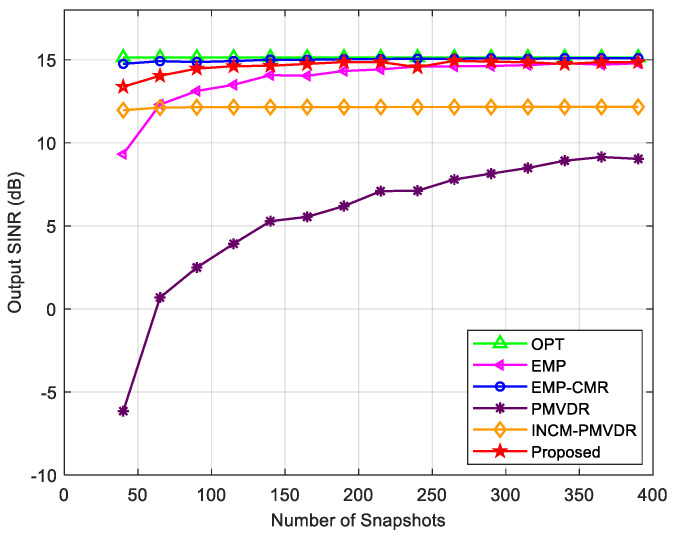
Output SINR versus the number of snapshots in the case of multiple mainlobe interferences.

In the next step, we analyzed the relationship between the output SINR of the proposed method and the number of snapshots. Figure 11 shows the variation in the output SINR of each method with the number of snapshots under multiple mainlobe interferences. The proposed method is basically not affected by the number of snapshots, has better performance than the INMC-PMVRD and PMVDR methods, and is close to the optimal output. The PMVDR method directly uses the sampling covariance matrix to calculate the beamforming weight vector. However, the estimation accuracy of the sampled covariance matrix is relatively low under the low snapshot conditions, which in turn leads to a decline in beamforming performance and a lower output SINR. INCM-PMVDR belongs to the reconstruction method and is basically not affected by the number of snapshots. But this method requires a reasonable division of angle intervals for the desired signal, mainlobe interferences, and sidelobe interferences, and its performance is constrained by the accuracy of angle sector division. The simulation results of the EMP and EMP-CMR methods shown in the figure are relatively ideal due to the direct use of the covariance matrix without the desired signal component, resulting in a stable output SINR.

## 5. Conclusions

This paper proposed a mainlobe interference suppression method for polarization-sensitive arrays. It utilizes the POL-SPICE algorithm for parameter estimation and then applies the results to the covariance matrix reconstruction process, improving the reconstruction accuracy. Under the condition of multiple mainlobe interferences, this method constructs an eigen-projection matrix by determining the eigenvectors corresponding to each mainlobe interference, thereby blocking the mainlobe interferences and enhancing the suppression performance against multiple mainlobe interferences. Then, SINCM reconstruction is performed to solve for the joint beamforming weight vector in the spatial–polarization domain, ultimately achieving effective suppression of main and side lobe interferences while preserving the shape of the mainlobe.

Compared with the existing methods, although the proposed method sacrifices some computation time, it achieves more accurate parameter estimation results and higher reconstruction accuracy of the INCM. It can handle received data containing the desired signal and multiple mainlobe interferences, exhibiting better interference suppression performance and stronger robustness. Future research will focus on the robustness design of the method under conditions such as parameter estimation errors and steering vector mismatch, optimize the algorithm procedure to reduce computational complexity, and ultimately exploit the advantages of polarization-sensitive arrays to improve the interference suppression capability.

## Figures and Tables

**Table 1:** Target and interference parameter settings in the case of one or multiple mainlobe interference scenarios.

Signal Source	DOA and Polarization Parameter (*θ*, *γ*)	SNR/INR (dB)
Target	$\left(0{}^{\circ},30{}^{\circ}\right)$	[0,15]
Jamming 1	$\left(3{}^{\circ},15{}^{\circ}\right)$	20
Jamming 2	$\left(-4{}^{\circ},40{}^{\circ}\right)$	20
Jamming 3	$\left(-40{}^{\circ},50{}^{\circ}\right)$	30
Jamming 4	$\left(50{}^{\circ},60{}^{\circ}\right)$	35

**Table 2:** Comparative analysis of computational complexity for different parameter estimation methods.

Algorithm	Computational Complexity	Algorithm	Computational Complexity
MUSIC	$O(NM^{2}+M^{3}+M^{2}L)$	Capon	$O(NM^{2}+M^{3}+M^{2}L)$
RD-MUSIC	$O(NM^{2}+M^{3}+8M^{2}L^{\prime })$	PM	$O(NM^{2}+K^{2}L+K^{3}+KML)$
POL-SPICE	$O(NM^{2}+M^{3}CL+MCL^{2})$		

## Data Availability

The data presented in this study are available on request from the corresponding author.
